# COVID-19 in Africa: preexisting immunity and HIV

**DOI:** 10.1097/QAD.0000000000003079

**Published:** 2021-11-03

**Authors:** Jumari Snyman, Eduard J. Sanders, Thumbi Ndung’u

**Affiliations:** a Africa Health Research Institute; b HIV Pathogenesis Programme, The Doris Duke Medical Research Institute, University of KwaZulu-Natal, Durban, South Africa; c Kenya Medical Research Institute-Wellcome Trust Research Programme, Kilifi, Kenya; d Centre for Tropical Medicine and Global Health, Nuffield Department of Medicine, University of Oxford, Oxford, UK; e Ragon Institute of Massachusetts General Hospital, Massachusetts Institute of Technology and Harvard University, Cambridge, Massachusetts, USA; f Division of Infection and Immunity, University College London, London, UK.

**Keywords:** co-infections, cross-reactivity, HIV, immunity, SARS-CoV-2

The true impact of SARS-CoV-2 in Africa, the continent that has most people with HIV (PWH), is still unclear. To date, just over 7.5 million cases and 189 000 deaths have been reported from sub-Saharan Africa. These numbers are far below those reported in high-income countries such as the USA and UK (https://africacdc.org/covid-19, accessed August 23, 2021). Several factors have been proposed for the geographic variation in the impact of the virus including differences in population age distribution, underlying host genetic and immunological mechanisms [1], environmental factors [2], limited testing capacity in resource-poor settings, and underreporting due to stigma [3,4]. Of particular importance is the impact of infectious comorbidities such as HIV, malaria, and tuberculosis. The impact of HIV on SARS-CoV-2 infection and disease course is of specific interest because HIV infection is lifelong and associated with generalized immunodeficiency, which may influence clinical outcomes [5]. Moreover, HIV may alter immune responses to some pathogens and vaccines, with implications for health, and for diagnostic or surveillance algorithms that rely on immune biomarkers.

In this issue of *AIDS*, Crowell *et al.*[6] retrospectively screened blood samples from cohort participants collected prior to the first known case of COVID-19 in Kenya for anti-SARS-CoV-2 nucleocapsid IgM, IgA, and/or IgG antibodies using a commercial kit. In total, 19 (3.3%) participants had detectable levels of total antibodies with no difference in antibody responses between participants with and without HIV. PWH in this study were all virally suppressed. Further investigations demonstrated seven of these participants had a prior positive test since June 2019, predating the COVID-19 pandemic. The authors therefore concluded that positive antibody responses could either be due to cross-reactivity or asymptomatic infections. This study highlights two important points: the effect preexposure to other human coronaviruses (HCoVs) might have on SARS-CoV-2 infection and disease course and the possibility of cross-reactivity when performing diagnostic testing or surveillance for SARS-CoV-2 using immunoassays.

The presumed lower morbidity and mortality in Africa may be multifactorial but so far, preexisting cross-reactive immunity from other HCoVs or other circulating viruses cannot be ruled out [7–10]. Preexisting IgG cross-reactivity to SARS-CoV-2 S and N proteins in individuals uninfected and unexposed to the SARS-CoV-2 has been widely reported [9,11,12]. Preexposure and cross-reactivity due to circulating HCoVs have been hypothesized to modify SARS-CoV-2 infection outcomes by decreasing disease severity. The latter could also partially explain the age distribution of COVID-19 disease severity, as HCoV infection rates are higher in children than in adults and correlates with relative protection from COVID-19 disease [13]. Understanding preexisting immunity will be critical to elucidate susceptibility to SARS-CoV-2 infection in Africa as well as the natural course of disease. Further, identifying conserved cross-reactive epitopes in HCoV or other mechanisms could prove beneficial for development of a universal vaccine or immune-based therapies.

On the contrary, cross-reactivity caused by seasonal coronaviruses may result in incorrect or false-positive results when immunoassays are used for diagnostics or sero-surveillence [14,15]. Several community-based and population-based sero-surveys in Africa have demonstrated seroprevalences ranging from 8.8% to 60.6% [8,16–18], sometimes much higher than reported case data would suggest to be the true prevalence. Interestingly, recent studies on the cross-reactivity of SARS-CoV-2 and other HCoVs demonstrated significantly higher cross-reactivity in sub-Saharan Africa compared to the USA [7,18]. Similar results were reported by Tso *et al.*[7] wherein a significant cross-reactivity with the N-protein was observed, although no differences were reported against the S protein. Anti-SARS-CoV-2 S1-RBD antibodies have also been shown to have a higher sensitivity versus N protein antibodies for both acute and postinfection phases with the anti-N IgG antibodies waning after acute infection [7,19]. These data suggest that immunoassays to estimate seroprevalence versus diagnostics may require fine-tuning, including careful consideration of viral protein targets [20].

Considering the high prevalence of HIV in some communities, particularly in south and eastern Africa, it is important to understand its potential implications for health, diagnostics, and surveillance related to the SARS-CoV-2 virus. Data suggest that individuals with advanced HIV, low CD4^+^ T-cell counts, and a high HIV viral load are more at risk for severe COVID-19 outcomes compared with antiretroviral therapy (ART) controlled HIV and that these individuals should be prioritized for SARS-CoV-2 vaccination [21–25]. However, studies have reported similar humoral immune responses between PWH on ART and HIV-uninfected individuals [26,27]. Given that individuals with suppressed HIV are less likely to suffer severe disease outcomes, focus should turn towards the impact that COVID-19 has on access to treatment and care for PWH, and the impact of non-ART controlled HIV infection on COVID-19 [23]. A recent global cross-sectional survey reported that 18% of MSM were either unable to refill or access their ART prescriptions [28] inevitably resulting in an increased viral load and low CD4^+^ T-cell count. Persistent SARS-CoV-2 infection has also been reported in immune-compromised individuals such as PWH who do not have ART-controlled infection. These individuals should be considered during sero-surveillance studies, as they may not reliably develop anti-SARS-CoV-2 antibody responses [29]. Therefore, implementing a combined use of molecular and serological testing will be necessary for continued surveillance [20]. A cause for concern is the increasing reports that prolonged viral replication and intrahost evolution in immunocompromised persons are contributing to the emergence of new variants capable of immune escape [30–34]. Superinfections in immunocompromised, persistently infected individuals have also been reported resulting in adverse clinical outcomes [35,36].

In conclusion, preexisting immunity to HCoVs may need to be further investigated as one potential mechanism underlying the lower prevalence and severity of SARS-CoV-2 infection in certain geographic areas in Africa. Further, comparative seroprevalence and serosurveillance studies that rely on immune assays need to be designed and interpreted with caution, considering the specific or unique epidemiological characteristics, particularly those related to HCoV population dynamics and SARS-CoV-2 persistent infections, as these factors may influence assay sensitivity and specificity within regions. A research gap exists in addressing the impact of infectious comorbidities on SARS-CoV-2 infection outcomes, particularly conditions prevalent in LMICs such as HIV. The impact of HIV on SARS-CoV-2 infection trajectory is incompletely understood but evidence suggests that uncontrolled HIV negatively impacts SARS-CoV-2 clinical outcomes, and the pandemic has impended healthcare access for PWH. COVID-19 vaccinations should therefore be prioritized for PWH especially for individuals with poorly controlled HIV, while African countries with low vaccination coverage should ameliorate vaccine campaigns.

## Acknowledgements

This work was supported through the Africa Health Research Institute (AHRI) Wellcome Trust Strategic Core Award Number 201433/A/16/A. This work was also supported through the Sub-Saharan African Network for TB/HIV Research Excellence, a Developing Excellence in Leadership, Training and Science (DELTAS) Africa Initiative [grant No. DEL-15–006]. The DELTAS Africa Initiative is an independent funding scheme of the African Academy of Sciences (AAS)'s Alliance for Accelerating Excellence in Science in Africa and supported by the New Partnership for Africa's Development (NEPAD) Planning and Coordinating Agency with funding from the Wellcome Trust (grant No. 107752/Z/15/Z) and the UK government.

### Conflicts of interest

There are no conflicts of interest.
